# Bringing *Planctomycetes* into pure culture

**DOI:** 10.3389/fmicb.2012.00405

**Published:** 2012-12-03

**Authors:** Olga M. Lage, Joana Bondoso

**Affiliations:** ^1^Department of Biology, Faculty of Sciences, University of PortoPorto, Portugal; ^2^CIMAR/CIIMAR – Interdisciplinary Centre for Marine and Environmental Research University of PortoPorto, Portugal

**Keywords:** *Planctomycetes*, isolation, *Rhodopirellula*, phylogeny, macroalgae biofilm, *Aquisphaera giovannonii*

## Abstract

*Planctomycetes* have been known since the description of *Planctomyces bekefii* by Gimesi at the beginning of the twentieth century (1924), although the first axenic cultures were only obtained in the 1970s. Since then, 11 genera with 14 species have been validly named and five candidatus genera belonging to the anaerobic ammonium oxidation, anammox bacteria have also been discovered. However, *Planctomycetes* diversity is much broader than these numbers indicate, as shown by environmental molecular studies. In recent years, the authors have attempted to isolate and cultivate additional strains of *Planctomycetes*. This paper provides a summary of the isolation work that was carried out to obtain in pure culture *Planctomycetes* from several environmental sources. The following strains of planctomycetes have been successfully isolated: two freshwater strains from the sediments of an aquarium, which were described as a new genus and species, *Aquisphaera giovannonii*; several *Rhodopirellula* strains from the sediments of a water treatment recycling tank of a marine fish farm; and more than 140 planctomycetes from the biofilm community of macroalgae. This collection comprises several novel taxa that are being characterized and described. Improvements in the isolation methodology were made in order to optimize and enlarge the number of *Planctomycetes* isolated from the macroalgae. The existence of an intimate and an important relationship between planctomycetes and macroalgae reported before by molecular studies is therefore supported by culture-dependent methods.

## THE *PLANCTOMYCETES*

*Planctomycetes* are a fascinating group of Bacteria, due to their unique and peculiar characteristics. Their distinct cell wall without the characteristic peptidoglycan, their nucleoid, their cell structure with compartmentalization, their reproduction, and their metabolism and ecological ubiquity make them an exciting group of organisms to study. The first planctomycete observed was morphologically similar to a planktonic fungus, *Planctomyces bekefii* (Gimesi, 1924), giving the name to this phylum. Curiously, it was never isolated in pure culture. The first report of the isolation of a planctomycete in axenic cultures is due to the work of James T. Staley (Staley, 1973). This organism was renamed taxonomically several times and was finally designated *Pirellula staleyi* (Schlesner and Hirsch, 1987). In 1986, a new order, *Planctomycetales*, and family, *Planctomycetaceae*, was proposed to accommodate the several members of this group that had been described, based on 16S rRNA gene sequence analysis and distinctive morphological characteristics (Schlesner and Stackebrandt, 1986). The phylum *Planctomycetes* was only proposed in 2001 (Garrity and Holt, 2001) and in 2006, a superphylum was designated to incorporate the phyla *Planctomycetes*, *Verrucomicrobia*, and *Chlamydiae* (superphylum PVC; Wagner and Horn, 2006). At present, the *Planctomycetes*, comprise 3 orders (*Planctomycetales*, *Phycisphaerales*, and “*Candidatus* Brocadiales”), 11 described genera and 14 species, and 5 *candidatus* genera with 14 *candidatus* species (Fukunaga et al., 2009; Jetten et al., 2010; Ward, 2010; Bondoso et al., 2011; Kulichevskaya et al., 2012). Although still small, these numbers are increasing. In the last 4 years, one new order, five new genera, and six species have been described. The cultured strains (see Schlesner, 1994; Wang et al., 2002; Pimentel-Elardo et al., 2003; Gade et al., 2004; Elshahed et al., 2007) are not at all representative of the great diversity and ubiquity that has been revealed by molecular microbial ecology techniques (Kirkpatrick et al., 2006; Penton et al., 2006; Schmid et al., 2007; Woebken et al., 2007; Lachnit et al., 2011; Pizzetti et al., 2011a; Pollet et al., 2011; Fuchsman et al., 2012; Ivanova and Dedysh, 2012). In fact, of the 11045 clone sequences belonging to *Planctomycetes* in Ribosomal Database Project (RDP; Cole et al., 2009) only 291 (2%) have been isolated in pure culture.

The remarkable characteristics of this group, together with the need to isolate new strains in pure cultures to extend our knowledge of their physiological role in microbial communities, prompted the authors to investigate *Planctomycetes*. During isolation experiments, methodological improvements were achieved and different environmental sources were attempted. In initial work, isolation of a single strain of *Planctomycetes* was achieved from the sediments of an ornamental freshwater aquarium. The bacteria obtained represent a novel genus within the *Planctomycetales* and was taxonomically described as *Aquisphaera giovannonii* (Bondoso et al., 2011). Subsequent work was focused on the marine environment and isolates were successfully obtained from the epiphytic community of macroalgae and from the sediment of a treatment water recycling tank of a marine fish farm. This work allowed the isolation of a collection of more than 150 *Planctomycetes* that represents new taxa that are being, or have already been, characterized.

## IMPROVEMENTS IN ISOLATION METHODOLOGIES

Knowledge of bacterial diversity and physiology was long based only on cultured organisms. With the golden era of the molecular revolution, the abundance, diversity, and ecology of microorganisms gained another dimension. This was especially relevant in marine aquatic environments, as was the case for the discovery of the oligotrophic ubiquitous bacterioplankton SAR11 cluster (Giovannoni et al., 1990). However, knowledge on certain aspects of the biology of microorganisms cannot be reached unless the organisms are available in culture. As an example, the cultivation of *Pelagibacter ubique*, a member of the SAR11 clade, by Rappé et al.(2002) enabled many unanswered questions to be addressed (Joint, 2008), such as the need of exogenous reduced sulfur compounds for a strain without the genes for assimilatory sulfate reduction.

For a longtime, only a few cultivable *Planctomycetes* were available and knowledge of this group was scarce. But several cultivation methods and media formulations for the isolation of planctomycetes were achieved in the last few decades (Schlesner, 1994; Zengler et al., 2002; Winkelmann and Harder, 2009). These advances were especially due to Dr. Heinz Schlesner’s work (Schlesner, 1994), leading to the isolation of a great number of isolates. More recently, several attempts have been made to bring “*Candidatus* Brocadiales” into culture. However, their isolation in pure culture has not succeeded. One main difficulty relies on their slow growth rate, with a doubling time of 10 days. After great technological accomplishments, they are, presently grown in membrane bioreactors with a purity of enrichment of 97.6% (van der Star et al., 2008; Kartal et al., 2010).

*Planctomycetes* are comparatively slow growing organisms with low demand for carbon and nitrogen sources. This makes them difficult to isolate in common media because they are easily outgrown by bacteria with faster growth rates. The utilization of media that have a relatively low content in yeast extract and peptone (usually less than 0.5%) and with the addition of glucose as a carbon source is useful for the isolation of these organisms. The addition of vitamin B12 to the isolation media, required by some members of the *Planctomycetes*, and micro- and macronutrients, also produced favorable results. The selective isolation of *Planctomycetes* is very much based on the capacity of these bacteria to grow in the presence of β-lactam antibiotics that affect peptidoglycan biosynthesis in dividing cells of the majority of bacteria (Schlesner, 1994). Some *Planctomycetes* are also resistant to the antibiotic streptomycin. The inhibition of bacterial growth by the action of these two antibiotics gives the relatively slow growing *Planctomycetes* the possibility to form colonies on the isolation plates. Another isolation strategy is to provide *N*-acetylglucosamine (NAG) as the only carbon and nitrogen source (Schlesner, 1994). Chitin composed of NAG monomers is the second most abundant organic compound in nature, and is present in fungi and several animals, namely copepods, that produce billions of tons of this compound annually (Yu et al., 1991). Ultimately, NAG ends up covering the ocean floor, where it is metabolized by bacteria. *Planctomycetes* are commonly present in marine sediments (Rusch et al., 2003; Musat et al., 2006; Chipman et al., 2010; Hu et al., 2010) where the availability of NAG favors the selection of the metabolic pathways for its degradation.

Besides the overgrowth of rapid growing bacteria, another problem, commonly faced when isolating bacteria from environmental samples, is the rapid and invasive growth of fungi. To inhibit fungal growth, cycloheximide or amphotericin B, are commonly added to the growth media (Schlesner, 1994; Wang et al., 2002; Winkelmann and Harder, 2009). However, these antifungal compounds have not always proven to be effective and fungicides like pevaryl (econazole nitrate; 1%) and benlate (benomyl, or methyl l-(butylcarbamoyl)-2-benzimidazolecarbamate; 4 mg∙mL^–^^1^) appear to be more adequate in inhibiting fungal growth (Lage and Bondoso, 2011). Another improvement for planctomycetes isolation from the surface of portions of macroalgae was to pre-wash them in a mixture of pevaryl and benlate before their introduction in the culture medium. With this, fungal overgrowth can be notably reduced. For the isolation of epiphytic planctomycetes from macroalgae diverse inocula, as the macerated macroalgae, the resuspended biofilm obtained from scrapping the surface of the macroalgae and the direct use of portions of the macroalgae on the isolation media were tested. This has enabled the isolation of a larger number of strains. These improvements, associated with the use of antibiotics (200 mg∙mL^–^^1^ ampicillin and 1000 mg∙mL^–^^1^ streptomycin), allowed the authors to obtain a large collection of culturable *Planctomycetes*, essentially from the surface of macroalgae (Lage and Bondoso, 2011).

## DIVERSITY OF *Rhodopirellula* ISOLATED FROM MARINE ENVIRONMENTS

The majority of *Planctomycetes* isolates recovered belong to the genus *Rhodopirellula. *These organisms are widespread and have been described in many marine environments, including brackish and marine water from the Kiel Fjord on the Baltic Sea (Schlesner et al., 2004), meso-eutrophic lakes (Pizzetti et al., 2011b), other European seas (Winkelmann and Harder, 2009; Pizzetti et al., 2011a), marine snow (DeLong et al., 1993; Vergin et al., 1998), diatom blooms in Oregon coastal waters (Morris et al., 2006), tissues of the Mediterranean sponge *Aplysina aerophoba* (Pimentel-Elardo et al., 2003; Gade et al., 2004), and the giant tiger prawn, *Penaeus monodon* (Fuerst et al., 1991) as well as the biofilm of *Laminaria hyperborea* (Bengtsson and Ovreas, 2010). However, the only taxonomically described organism is *Pirellula* sp. strain 1 (presently *Rhodopirellula baltica* SH1^T^), isolated from the water column of the Kiel Fjord as a free-living bacterium (Schlesner, 1994; Schlesner et al., 2004). The great heterogeneity within the genus *Pirellula* led Schlesner et al. (2004) to emend this genus and create two new genera, *Rhodopirellula* and *Blastopirellula*. The 16S rRNA gene sequence analysis, DNA–DNA hybridization and MLSA analysis (Schlesner et al., 2004; Winkelmann et al., 2010) revealed that *R. baltica* is a cosmopolitan species with a great genetic diversity.

**FIGURE 1:**
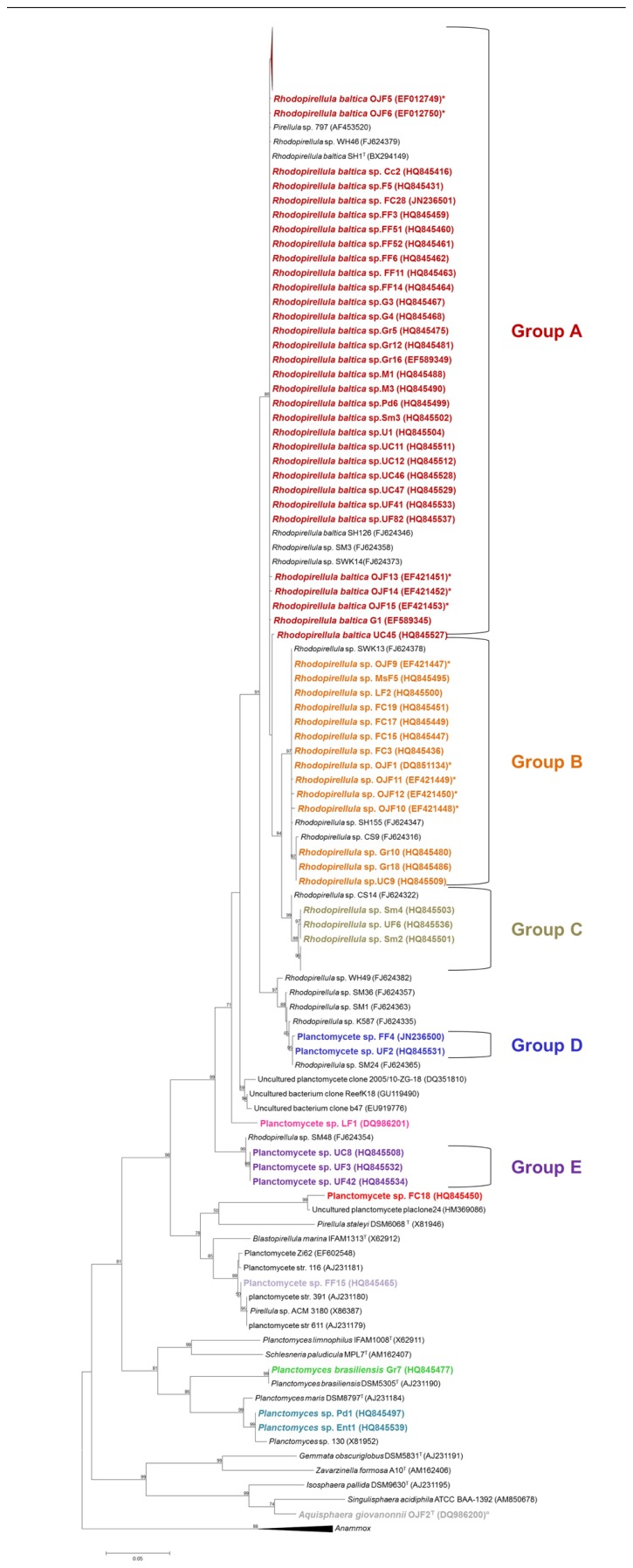
**Phylogenetic 16S rRNA gene tree generated by a maximum-likelihood analysis based on the Jukes–Cantor model, indicating the relationship of the isolates reported in this article (shown in bold) to members of the *Planctomycetes***. GenBank accession numbers are shown in parentheses. The *Anammox* genera were used as an outgroup. The numbers shown beside nodes are the percentages for bootstrap analyses; only values above 50% are shown. *Strains isolated from the fish farm sediments; ^¤^Novel genera isolated from an ornamental freshwater aquarium. Scale bar = 0.05 substitutions per 100 nucleotides.

The isolation experiments, both from the biofilm community of macroalgae (Lage and Bondoso, 2011) and the marine fish farm environments (Lage et al., 2012), revealed that the mono-speciated *Rhodopirellula* genus was dominant among the isolates and was clearly diverse (**Figure 1**). The biofilm community of the macroalgae sampled provided a total of 138 *Planctomycetes* isolates. The genus *Rhodopirellula* represented 92% of the total isolates, of which 71% are strains (more than 99.5% 16S rRNA gene similarity) within the species *R. baltica* (group A). The remaining 29% are subdivided in two groups (groups B and C), with about 97–98% of similarity in the 16S rRNA gene to the type species. *Rhodopirellula* sp. was also present in the sediment of a water treatment recycling tank of a marine fish farm, from which eleven isolates were obtained representing two main phylogenetic clusters as defined by the 16S rRNA gene sequence similarity. Six of the isolates are intimately related to *Rhodopirellula baltica* (in group A) with more than a 99% 16S rRNA gene sequence similarity and the other five isolates, with a 97.7–97.9% 16S rRNA gene sequence similarity to this species, belong to group B. In total, 149 isolates within the genus *Rhodopirellula* from the two marine environments of the north Atlantic coast of Portugal were obtained in pure culture. These results confirmed the presence of this genus in the biofilm community of ten of the three major macroalgae groups (*Gelidium pulchellum*, *Chondrus crispus*, *Corallina* sp.,* Gracilaria bursa-pastoris*, *Grateloupia turuturu*, *Fucus spiralis*, *Mastocarpus stellatus*, *Porphyra dioica*, *Sargassum muticum*, and *Ulva* sp.) and in marine fish farm environments. The *Rhodopirellula* sp. groups B and C are less numerous than *R. baltica*. Group B was isolated from the sediment of a treatment water recycling tank of a marine fish farm, from five macroalgae (*G. bursa-pastoris*, *F. spiralis*, *Laminaria* sp.,* M. stellatus*, and *Ulva* sp.), from Sweden (from algae surface), the Baltic Sea and sediments from Italy and Mallorca (Winkelmann and Harder, 2009). Group C was mainly isolated from macroalgae (*C. crispus*, *Corallina* sp., *G. bursa-pastoris*, *F. spiralis*, *M. stellatus*, *S. muticum*, and *Ulva* sp.) sampled on the Carreço rocky beach in the north cost of Portugal and two more isolates from sediments in Brest, France (Winkelmann and Harder, 2009). So far, culture-independent methods did not detect the presence of this group in other habitats or sources.

Presently, the RDP (Cole et al., 2009) accounts for 593 sequences classified in the genus *Rhodopirellula*, of which 416 are from uncultured organisms. Even though only one species was described in the genus *Rhodopirellula*, intrageneric diversity points to the existence of new species in this genus. An ERIC-PCR fingerprinting study performed with the strains isolated from the fish farm environment revealed a great intraspecies genetic variability. It was possible to differentiate 9 genotypes within the 11 isolates (Lage et al., 2012). This was also observed by Winkelmann et al. (2010) by multi-locus typing and Box-PCR and by Schlesner et al. (2004) by DNA-DNA hybridization.

## *PLANCTOMYCETES* DIVERSITY HIDDEN IN MACROALGAE BIOFILMS

Other taxa of *Planctomycetes*, in addition to *Rhodopirellula*, were isolated from the biofilm community of macroalgae (**Figure 1**). Three potential new genera (groups D, E and strain LF1) are phylogenetically more closely related to *R. baltica* (93–96% 16S rRNA gene sequence similarity), forming three clear separate clusters within the *Planctomycetales*. One isolate (strain FF15) is related to *Blastopirellula marina* (95% 16S rRNA gene sequence similarity) and isolate FC18 is phylogenetically more closely related to *Pirellula staleyi* (85.4% similarity in the 16S rRNA gene sequence) and shares an 87% 16S rRNA gene sequence similarity to *B. marina* and *R. baltica*. Both represent potential new genera isolated from *Fucus spiralis*. Strains Pd1 and UiF1 (isolated, respectively, from *Porphyra dioica* and *Ulva intestinalis*) sharea 98% 16S rRNA gene sequence similarity to *P. maris*. Curiously, one strain from *G. bursa-pastoris*, Gr7, which has 100% similarity to *Planctomyces brasiliensis* DSM5305^T^ from Lagoa Vermelha, a salt pit environment from Brazil (Schlesner, 1989), was also isolated. Besides the three OTUs related to *Rhodopirellula*, the diversity of *Planctomycetes* associated with macroalgae represents more seven different OTUs based on a 98% cut-off (Stackebrandt and Ebers, 2006).

Macroalgae harbor a great diversity of *Planctomycetes* in their biofilm community. The relationship between *Planctomycetes* and macroalgae had been unveiled by Bengtsson and Ovreas (2010) and Bengtsson et al. (2010), who found that *Planctomycetes* account for 51–53% of the bacterial biofilm cells in July and September and 24% in February in the kelp *Laminaria hyperborean* from southwestern Norway. Clone libraries of the kelp revealed 23 OTUs at 98% sequence similarity, with the majority being species of *Rhodopirellula* and *Blastopirellula*. Lachnit et al. (2011) also found a temporal fluctuation of *Planctomycetes* in association with *Fucus vesiculosus*, *Gracilaria vermiculophylla*, and *Ulva intestinalis. *The denaturing gradient gel electrophoresis (DGGE) bands were related to *Rhodopirellula*, *Planctomyces*, and *Blastopirellula*. *Planctomycetes* were also found in *Ulva australis* and accounted for 3.4% of the total clones (Burke et al., 2011). A new order of *Planctomycetes*, *Phycisphaerales*, was proposed to accommodate a novel genus isolated from the surface of *Porphyra* sp., *Phycisphaera* (Fukunaga et al., 2009). These studies, together with our results (Lage and Bondoso, 2011), show that macroalgae provide a suitable eutrophic environment that supports the growth of heterotrophic *Planctomycetes*. Macroalgae produce organic sulfur compounds (Michel et al., 2006) and excrete photosynthetically derived dissolved exudates. *Planctomycetes* colonies recovered from the surface of small portions of macroalgae (Lage and Bondoso, 2011) proved that the growth of *Planctomycetes* can be supported by macroalgae compounds. Further support for the hypothesis of a nutritional role of macroalgae for *Planctomycetes* comes from growth experiments (Lage and Bondoso, 2011). Water-soluble extracts of *Ulva* sp. and *F. spiralis* enabled the growth of some *Planctomycetes*. However, in an enrichment study that used kelp constituent carbon sources to support the cultivation of bacterial populations associated with the kelp *Laminaria hyperborean*, *Planctomycetes*, and *Verrucomicrobia*, the two most frequently detected bacterial lineages, were not identified among the cultured bacteria (Bengtsson et al., 2011). Nevertheless, *Planctomycetes* are able to grow on several carbohydrates (mono and disaccharides and complex ones) as a carbon source and on the chitin monomer NAG (Schlesner, 1994; Rabus et al., 2002). *R. baltica* possesses several genes encoding for 110 sulfatases (Glöckner et al.,2003) and presumably could be involved in the recycling of carbon from complex sulfated heteropolysaccharides (Wecker et al., 2009). Macroalgae may benefit by hosting *Planctomycetes* biofilm communities through competitive exclusion of potentially undesirable microbes, the production of secondary metabolites and a role in avoiding desiccation, as *Planctomycetes* can possess extracellular matrices.

## CONCLUSION

*Planctomycetes* are far from being a well-known group of Bacteria. Although their first observation goes back to the beginning of the last century, it is only since the 1970–1980s that more regular publications began to appear. In this mini review, culture aspects of this group have been highlighted, providing a summary of the work developed over recent years with the aim of isolating in pure culture *Planctomycetes* from several environmental sources. Methodological improvements through the use of a new combination of fungicides and antibiotics, the use of low organic media and portions of macroalgae that were essential for successful isolations have been pointed out. A large collection of *Planctomycetes* was, thus, obtained from the biofilm community of macroalgae, from the sediments of the water treatment tank of a fish farm, as well as from the sediments of a freshwater aquarium. As some of the isolates are novel taxa, their description is imperative. With these characterizations, knowledge on the morphology, metabolism, and ecology of this group will be enlarged. This phylogenetic diversity is the base for future ecological work namely on the comprehension of the interaction macroalgae–*Planctomycetes* and on the application of molecular approaches to the study of the biogeographic distribution of *Planctomycetes* in coastal environments. With this work the frontiers of diversity are being pushed forward, as indeed they should be.

## Conflict of Interest Statement

The authors declare that the research was conducted in the absence of any commercial or financial relationships that could be construed as a potential conflict of interest.
